# Supplementary material for: Deep microbial proliferation at the basalt interface in 33.5–104 million-year-old oceanic crust
Source: Commun Biol. 2020 Apr 2;3:136. doi: 10.1038/s42003-020-0860-1 (PMC7118141; doi:10.1038/s42003-020-0860-1)
Supplement: Supplementary file 1 — Supplementary Information [file 42003_2020_860_MOESM1_ESM.docx]

**Supplementary Information**

The data supporting the findings of this study are available within the paper and its Supplementary Information.


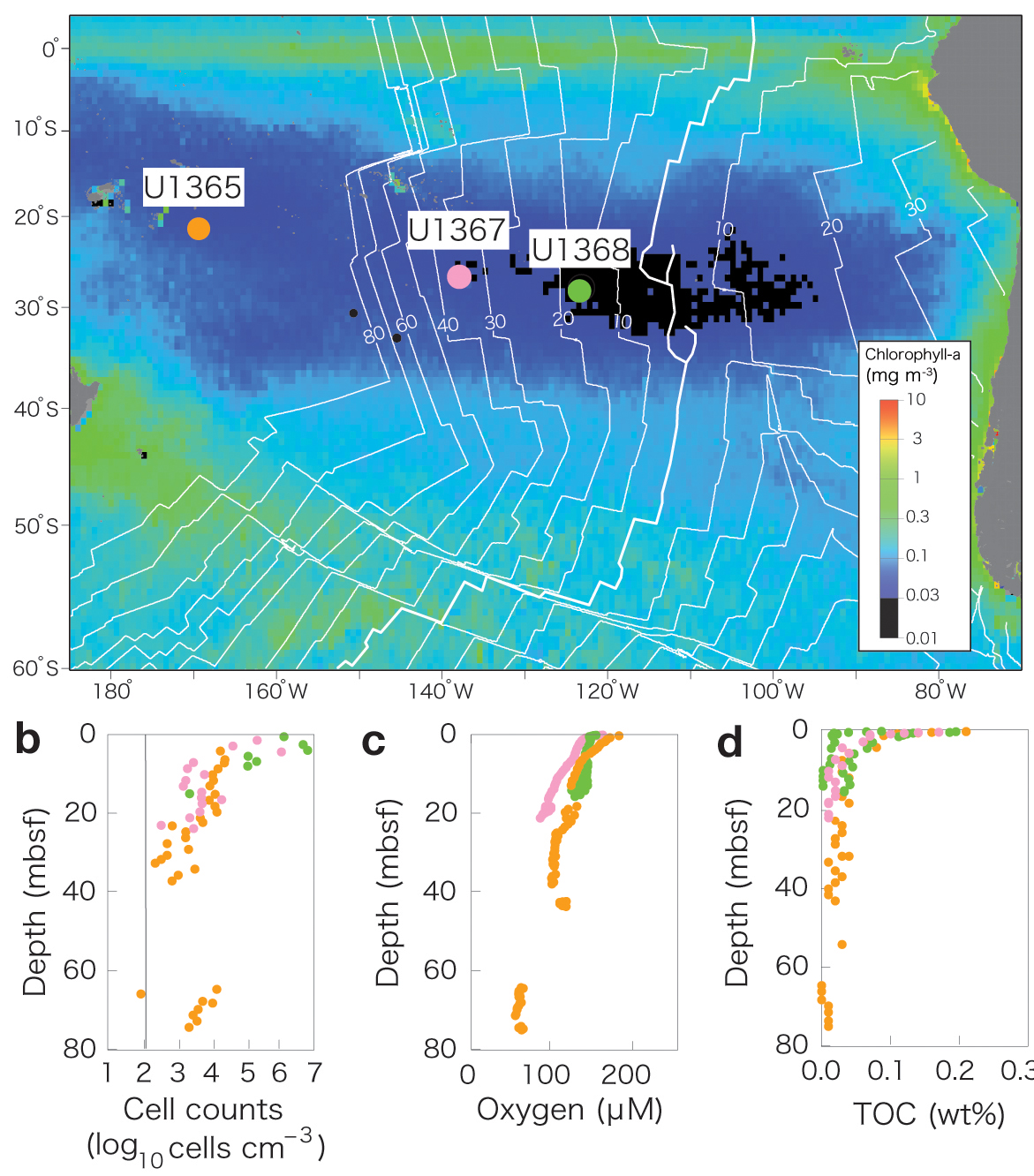


**Supplementary Figure 1| Biogeochemical characteristics of surface seawater and sediment overlying region of the drilled basaltic basement.** (**a**) Map of annual chlorophyll-a concentration in surface seawater. Sedimentary profiles of cell abundance (**b**), dissolved O_2_ (**c**), and total organic carbon (**d**). Profiles span the sediment column from seafloor to basement. The vertical line in (**b**) marks the minimum quantification limit.

**
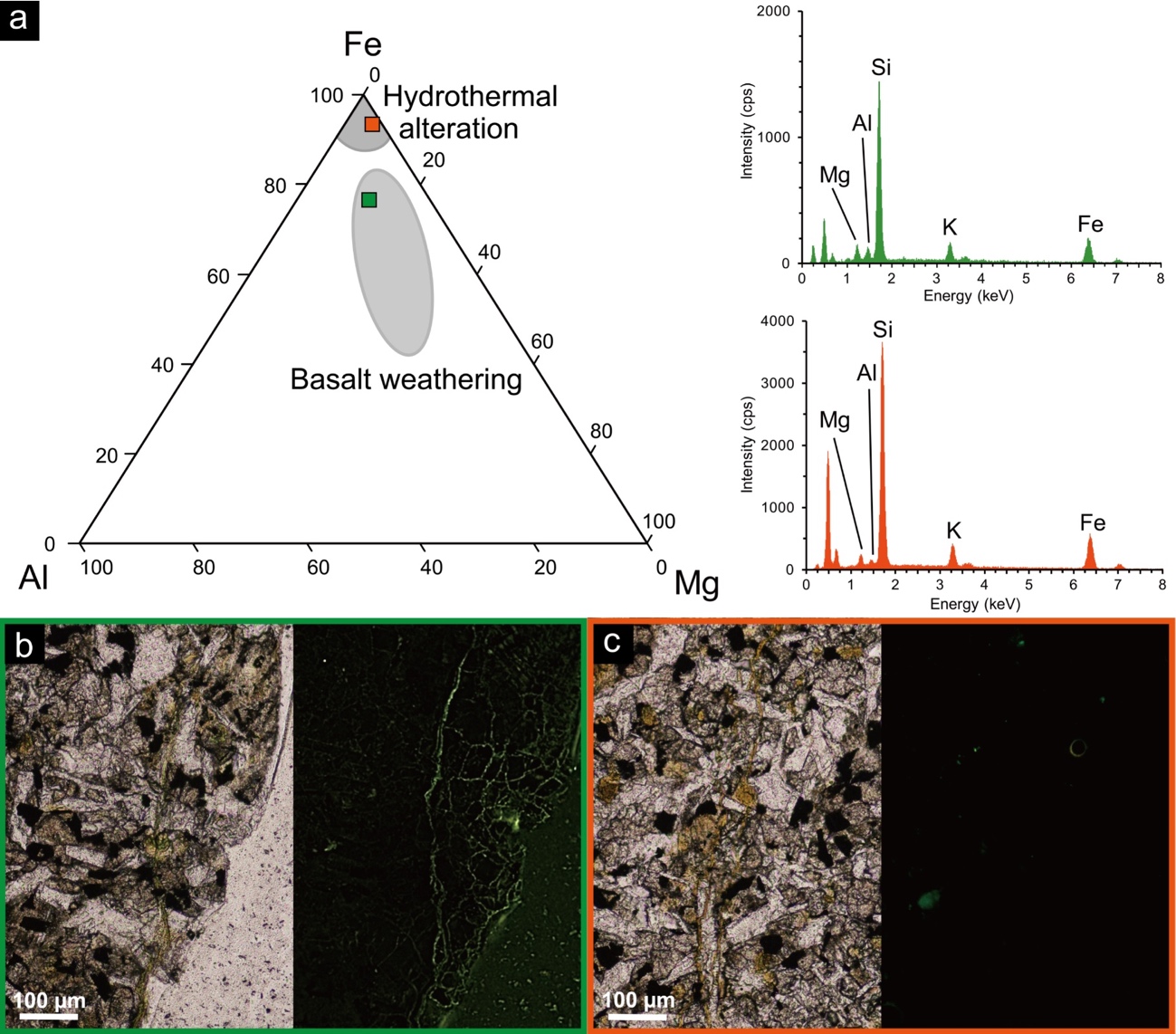
**

**Supplementary Figure 2|** **Chemical** **compositions of two types of Fe-rich smectite found in U1367F-6R1 and cell distributions revealed by staining of thin sections with SYBR-Green I.** (**a**) Trigonal diagram of Al-Fe-Mg content in sheet layer of nontronite and EDS spectra of nontronite formed by basalt weathering in green and hydrothermal alteration in orange. Fluorescence microscopy images of SYBR Green I-stained microbial cells with nontronite formed by basalt weathering (**b**) and hydrothermal alteration (**c**).


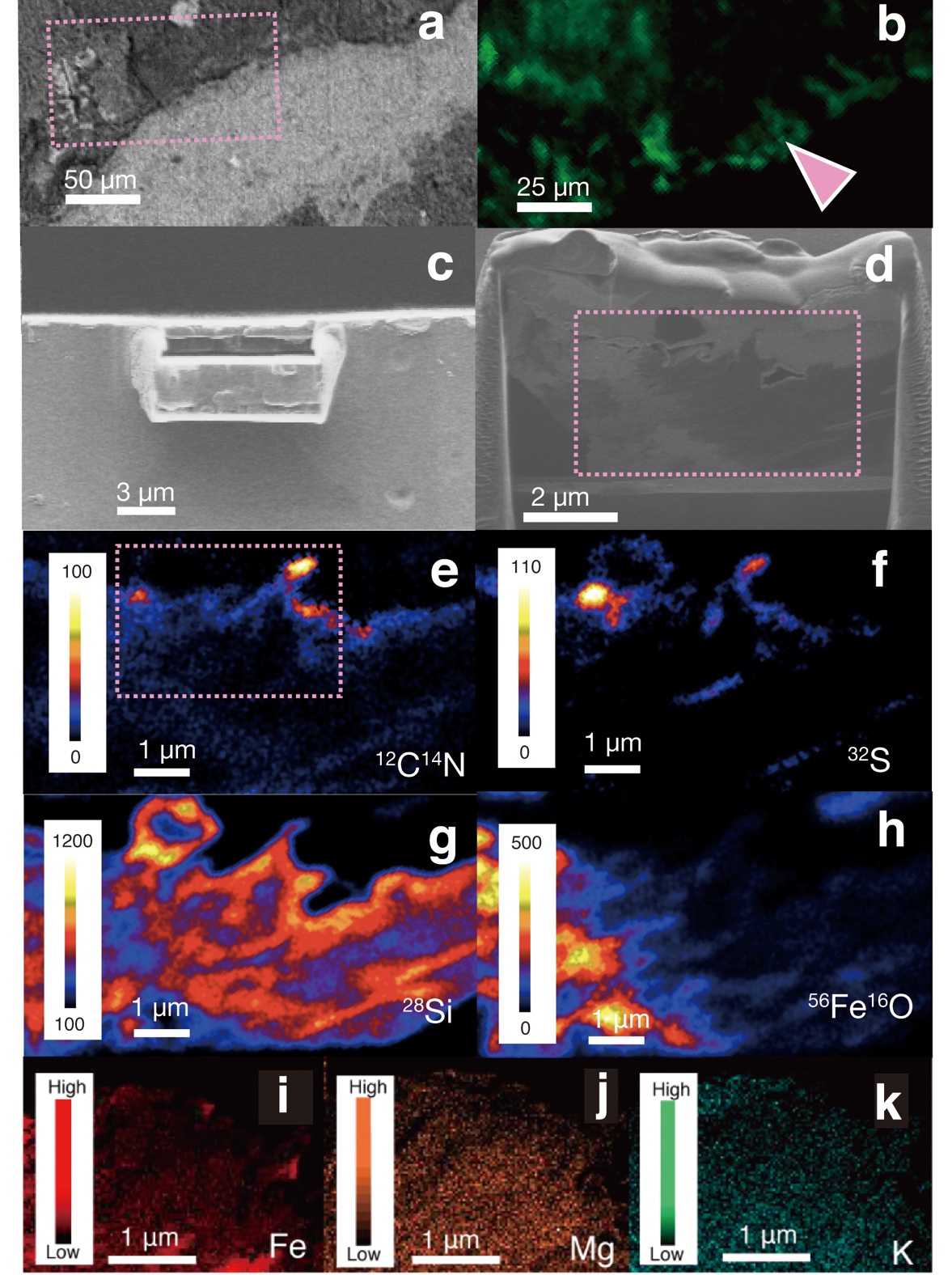


**Supplementary Figure 3|** **Single-cell characterizations of fracture-hosted microbial populations.** Scanning electron microscopy (SEM) image of the mineral-filled fractures in U1365E-12R2 (**a**). Confocal laser microscopy image of SYBR Gr­­een I-stained microbial cells (**b**). Ga ion image of a FIB thin section of U1365-12R2 (3-μm thick and a square region of ~10 × ~10 μm^2^) analysed using JAMSTEC NanoSIMS (**c**, **d**) and images of ^12^C^14^N^−^ (**e**), ^32^S^−^ (**f**), ^28^Si^−^ (**g**), and ^56^Fe^16^O^−^ (**h**) with intensity colour contours. STEM X-ray elemental mapping images of Fe (**i**), Mg (**j**), and K (**k**). Pink arrows and dashed rectangles denote spots or regions described in subsequent figures.


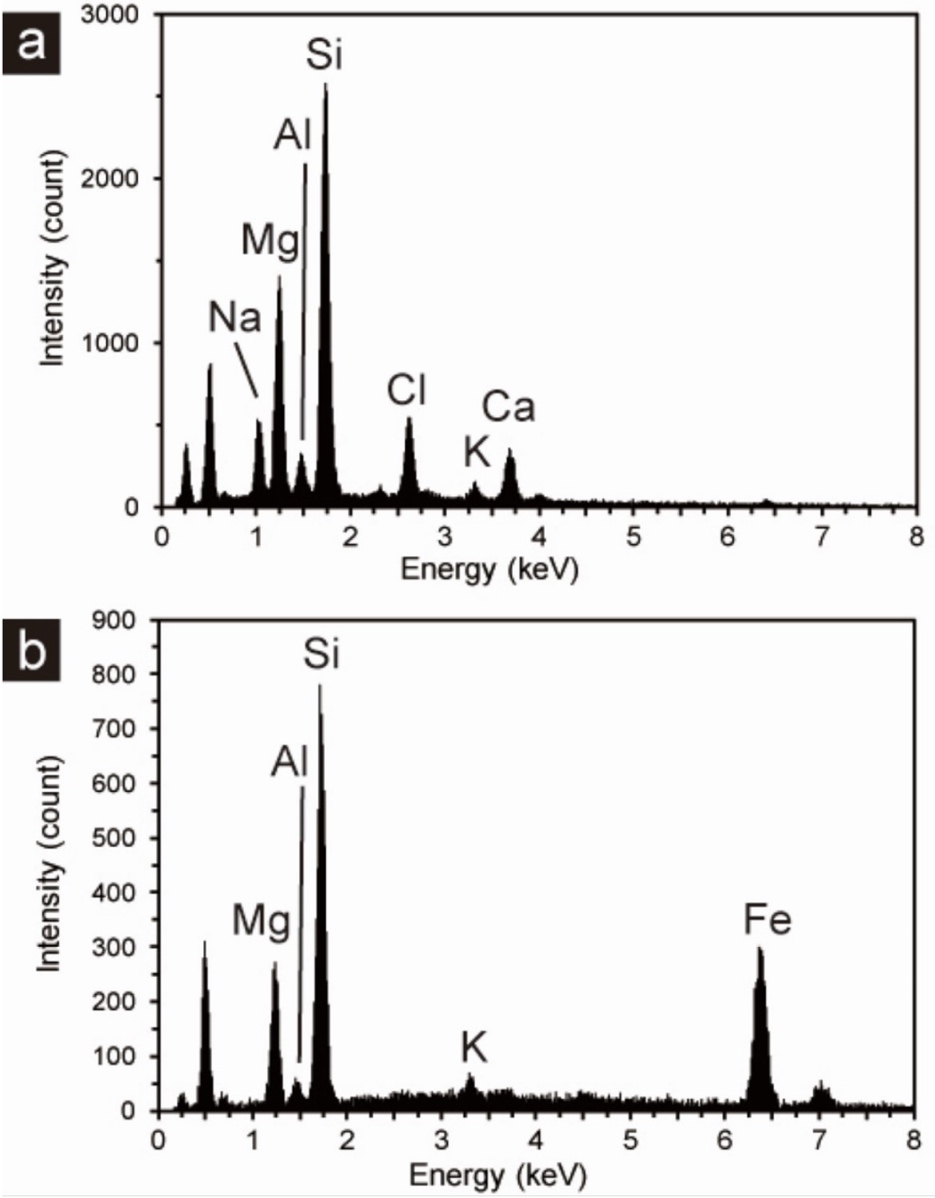
**Supplementary Figure 4|** **Chemical comparison of bentonite clay used for drilling fluid and nontronite spatially associated with microbial cells**. SEM-EDS spectra obtained from bentonite clay (**a**) and from nontronite (**b**).


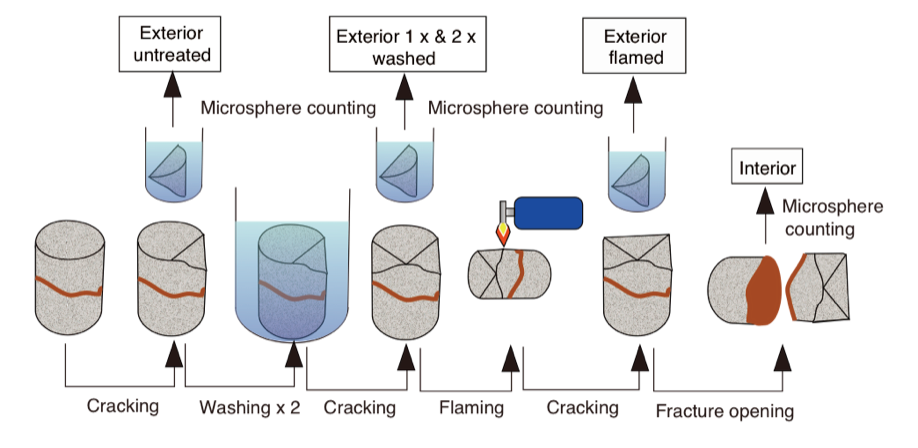
**Supplementary Figure 5| Schematic diagram of procedures undertaken for contamination evaluation and decontamination of drilling fluid.** Microsphere counting was performed for each step by epifluorescence microscopy.


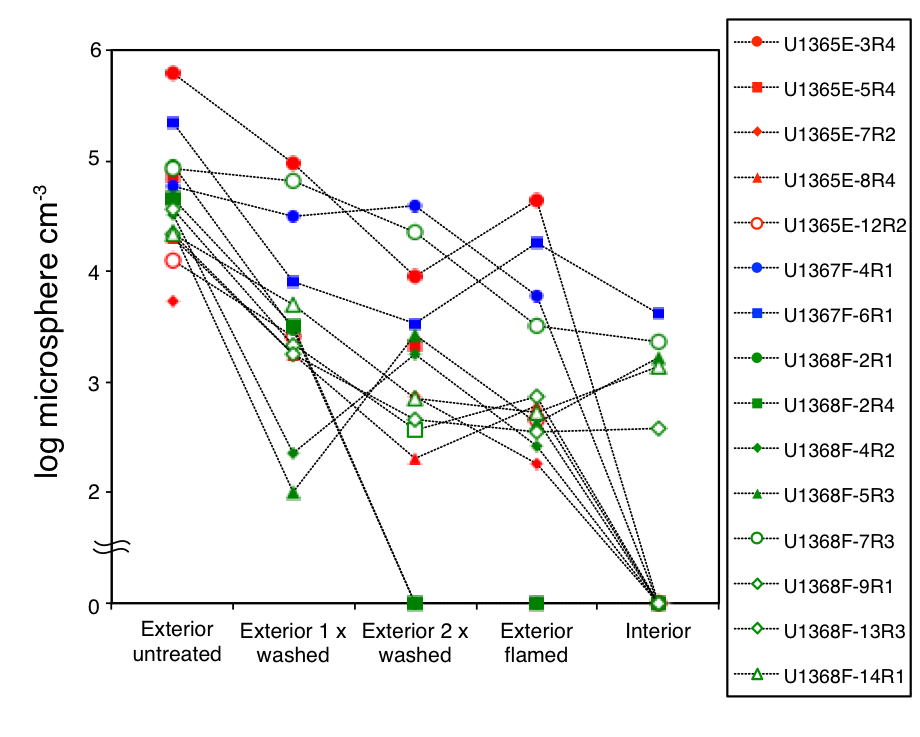
**Supplementary Figure 6| Effects of decontamination processes by washing and flaming.** Microscopic counts of fluorescence microspheres in basaltic core samples after each round of decontamination. Rock pieces are represented in cm^−3^, and microsphere density in the surface wash solutions was calculated from the core volume subjected to NaCl washing.

**Supplementary Figure 7| Phylogenetic distributions of the highly contaminated sample U1368F-7R3, the drilling-fluid sample from U1365E, and the negative control used for laboratory manipulations based on 16S rRNA gene sequences.** Colours and legends represent differences in taxonomic classification ranging from genus to phylum. Each proteobacterial class or phylum is shown in parentheses.


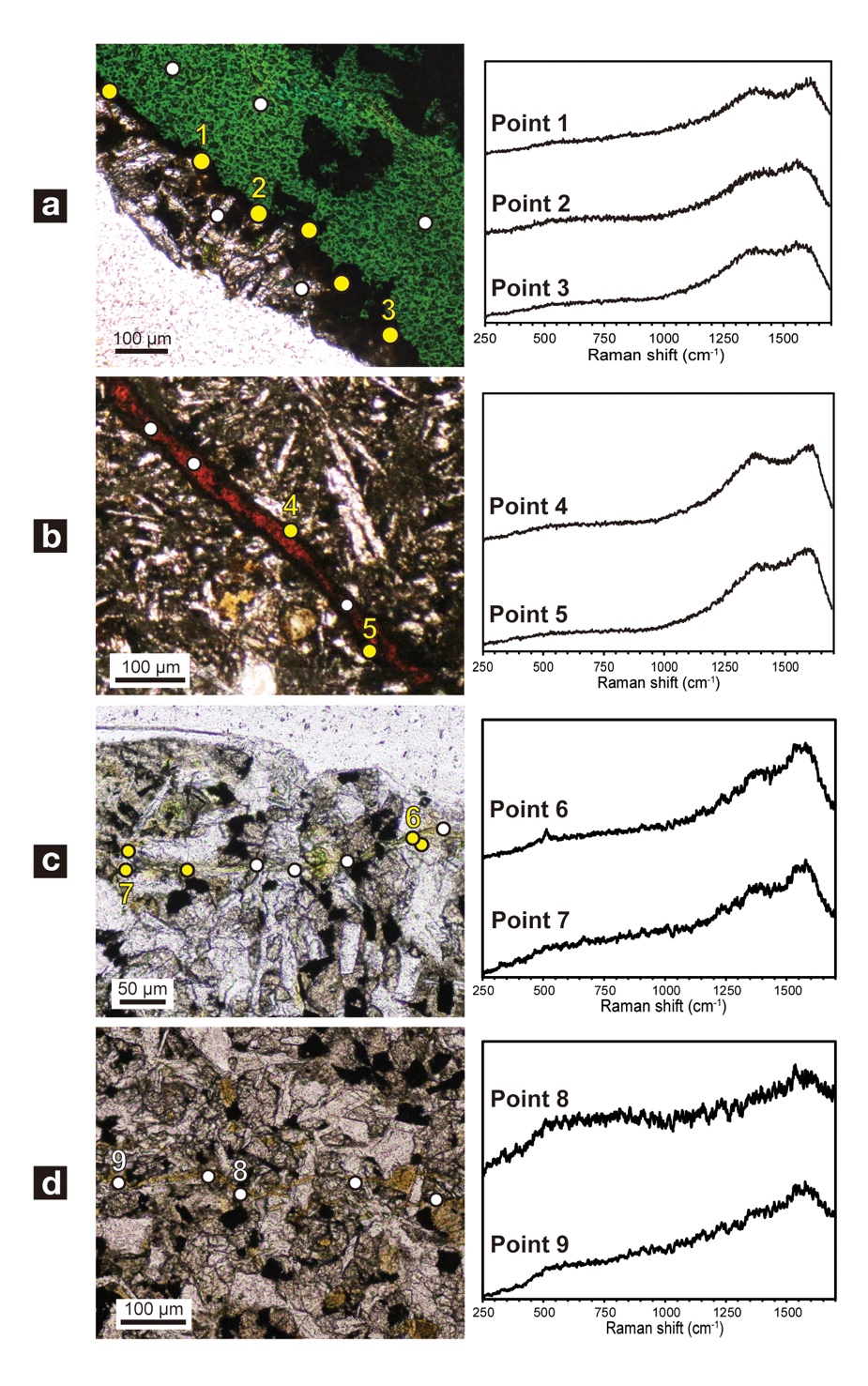


**Supplementary Figure 8| Raman spectra of microbe-nontronite assemblages in fractures/veins of basaltic basement.** Broad peaks at 1200–1600 cm^-1^ attributed to amorphous organic matter and a slope increasing with Raman shift attributed to smectite were obtained at the basalt interface in U1365E-8R4 (**a**), U1365E-12R2 (**b**), and U1367F-6R1 (**c**). Weak broad peaks at 1200–1600 cm^-1^ attributed to amorphous organic matter at Mg-poor nontronite in U1367F-6R1 (**d**). Yellow dots show points where strong peaks were obtained at 1200–1600 cm^-1^. White dots show points where weak peaks were obtained at 1200–1600 cm^-1^.

**
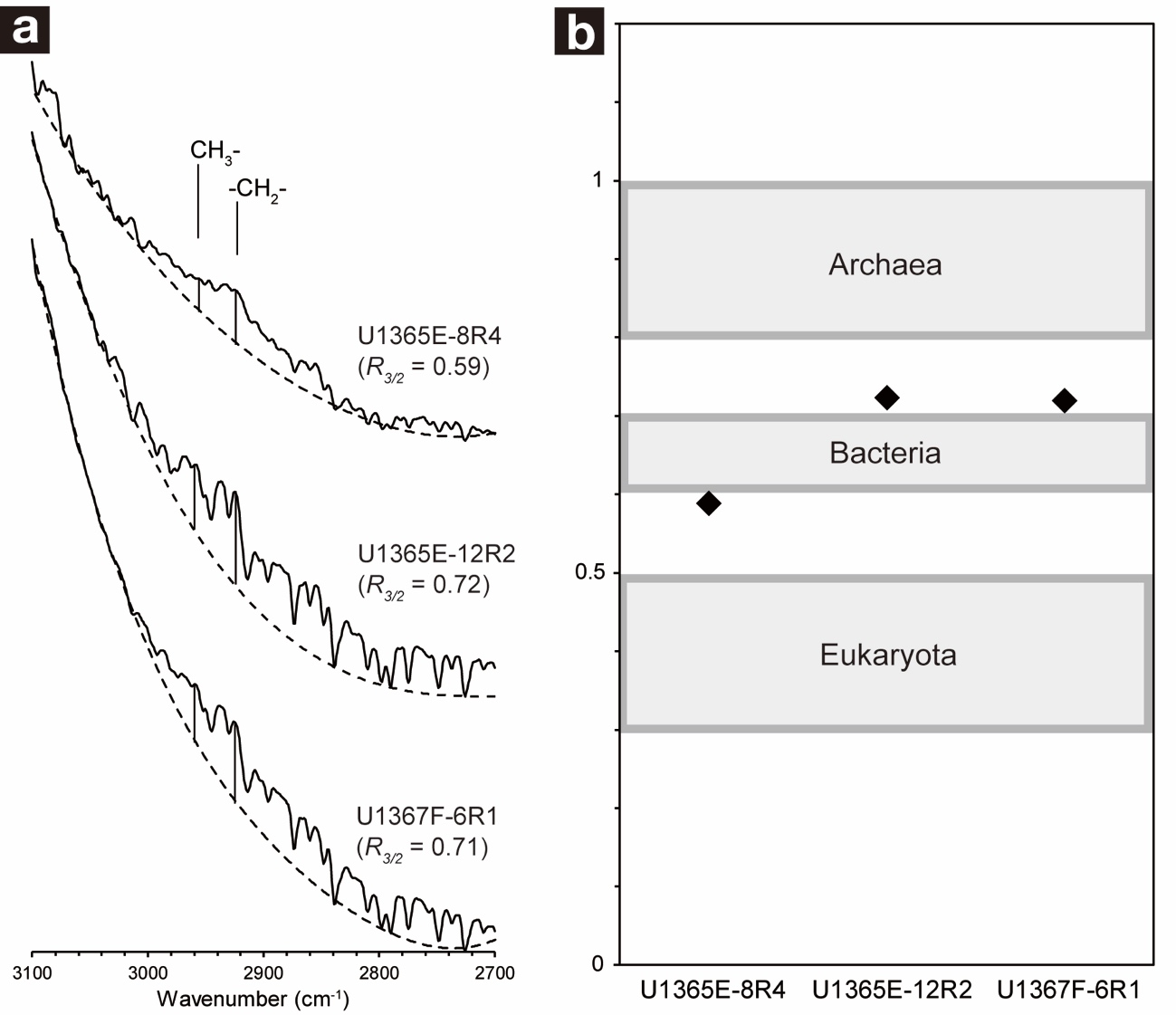
**

**Supplementary Figure 9|** **Domain-level lipid characteristics.** Lipid *R_3/2_* values calculated from FT-IR spectra of lipids of microbial cells in clay fractions collected from basaltic core samples of U1365E-8R4, U1365E-12R2, and U1367F-6R1 (**a**). Plots of Lipid *R_3/2_* values and the *R_3/2_* ranges of Archaea, Bacteria, and Eukaryotes.

**Supplementary Table 1|** Drilling site characteristics investigated for basaltic basement during IODP Expedition 329.

| Site | Water depth  (m) | Location | Total penetration depth  (mbsf) | Age  (Ma) | Sediment depth  (mbsf) | Basalt core recovery  (%) | Bottom sediment O_2_  (μM) | Bottom sediment nitrate  (μM) | Chlorophyll-a  (mg/L) |
| --- | --- | --- | --- | --- | --- | --- | --- | --- | --- |
|  |  |  |  |  |  |  |  |  |  |
| U1365 | 5697 | 23.8508°S, 165.6442°W | 124.2 | 104 | 71 | 74.6 | 120 | 39 | 0.03–0.10 |
| U1367 | 4284 | 26.4816°S, 137.9394°W | 55.5 | 33.5 | 17 | 11.2 | 162 | 40 | 0.03–0.10 |
| U1368 | 3738 | 27.9167°S, 123.1609°W | 115.1 | 13.5 | 11.8 | 27.6 | 187 | 41 | 0.01–0.03 |

mbsf: meters below the sea floor; NS: no sample was taken.

**Supplementary Table 2|** Chemical compositions of nontronite formed by hydrothermal alteration at Site U1367 and bentonite clay used for drilling mud.

| Site | U1367 | U1367 | U1367 | U1367 | U1367 | U1367 | U1367 |  | bentonite | bentonite |
| --- | --- | --- | --- | --- | --- | --- | --- | --- | --- | --- |
| Core Unit | 6R1 | 6R1 | 6R1 | 6R1 | 6R1 | 6R1 | 6R1 |  | 01 | 02 |
| SiO2 | 49.36 | 54.31 | 47.49 | 51.90 | 48.82 | 48.71 | 39.29 |  | 54.55 | 64.58 |
| Al2O3 | 1.08 | 1.28 | 0.68 | 1.54 | 0.81 | 0.91 | 1.29 |  | 12.42 | 23.68 |
| TiO2 | 0.17 | 0.00 | 0.31 | 0.37 | 2.46 | 0.42 | 0.66 |  | 0.00 | 0.08 |
| Fe2O3 | 41.18 | 36.27 | 44.03 | 37.09 | 40.93 | 41.30 | 51.72 |  | 3.09 | 0.00 |
| MnO | 0.00 | 0.00 | 0.00 | 0.00 | 0.00 | 0.00 | 0.00 |  | 0.00 | 0.00 |
| MgO | 2.34 | 2.67 | 1.77 | 2.88 | 1.43 | 2.16 | 0.74 |  | 18.85 | 0.49 |
| CaO | 1.17 | 0.61 | 1.50 | 1.25 | 1.06 | 1.15 | 1.38 |  | 3.71 | 2.87 |
| K2O | 4.45 | 4.58 | 4.21 | 4.57 | 4.39 | 5.25 | 4.92 |  | 4.18 | 3.78 |
| Na2O | 0.25 | 0.27 | 0.00 | 0.40 | 0.12 | 0.10 | 0.00 |  | 3.21 | 4.52 |
| sum | 100.00 | 100.00 | 100.00 | 100.00 | 100.00 | 100.00 | 100.00 |  | 100.00 | 100.00 |

**Supplementary Table 3|** List of basaltic core samples investigated in this study. Microsphere counts were obtained by epifluorescence microscopy observations.

| Site | Hole | Core | Type | Section | Depth  (mbsf) | Microsphere counts (Log_10_ microsphere cm^−3^) | | | | |
| --- | --- | --- | --- | --- | --- | --- | --- | --- | --- | --- |
|  |  |  |  |  |  | Untreated core | 1 st Exterior washed | 2nd Exterior washed | Exterior flamed | Interior |
| U1365 | E | 3 | R | 4 | 85.3 | 5.8 | 5.0 | 4.0 | 4.6 | ND |
| U1365 | E | 5 | R | 4 | 94.2 | 4.9 | NS | 3.3 | NS | ND |
| U1365 | E | 7 | R | 2 | 101.1 | 3.7 | NS | 2.3 | 2.3 | ND |
| U1365 | E | 8 | R | 4 | 109.6 | 4.3 | 3.3 | 2.3 | 2.8 | ND |
| U1365 | E | 12 | R | 2 | 121.8 | 4.1 | 3.4 | NS | 2.7 | ND |
| U1367 | F | 4 | R | 1 | 36.6 | 4.8 | 4.5 | 4.6 | 3.8 | ND |
| U1367 | F | 6 | R | 1 | 51.2 | 5.4 | 3.9 | 3.5 | 4.3 | 3.6 |
| U1368 | F | 2 | R | 1 | 9.7 | 5.0 | 3.5 | ND | ND | ND |
| U1368 | F | 2 | R | 4 | 13.4 | 4.7 | 3.5 | ND | ND | ND |
| U1368 | F | 4 | R | 2 | 30.0 | 4.5 | 2.4 | 3.3 | 2.4 | ND |
| U1368 | F | 5 | R | 3 | 40.2 | 4.4 | ND | 3.4 | 2.6 | 3.2 |
| U1368 | F | 7 | R | 3 | 50.4 | 4.9 | 4.8 | 4.4 | 3.5 | 3.4 |
| U1368 | F | 9 | R | 1 | 62.8 | 4.6 | 3.3 | 2.6 | 2.9 | ND |
| U1368 | F | 13 | R | 3 | 99.7 | 4.3 | 3.3 | 2.7 | 2.5 | 2.6 |
| U1368 | F | 14 | R | 1 | 106.1 | 4.3 | 3.7 | 2.9 | 2.7 | 1.1 |

mbsf: meters below the sea floor; ND: no cells or microspheres were observed by microscopic observations; NS: no sample was taken.

**Supplementary Table 4|** Comparison of total organic carbon concentrations between bulk and clay fraction.

|  | Total carbon concentration (%) | |
| --- | --- | --- |
| Sample ID | Bulk | Clay fractions |
| U1365E-8R4 | 0.036 | 0.123 |
| U1365E-12R2 | 0.012 | 0.111 |
| U1367F-6R1 | 0.015 | 0.329 |
