# Supplementary material for: Deep microbial proliferation at the basalt interface in 33.5–104 million-year-old oceanic crust
Source: Commun Biol. 2020 Apr 2;3:136. doi: 10.1038/s42003-020-0860-1 (PMC7118141; doi:10.1038/s42003-020-0860-1)
Supplement: Supplementary file 4 — Reporting Summary [file 42003_2020_860_MOESM4_ESM.pdf]

nature research

Corresponding author(s): Yohey Suzuki

Last updated by author(s): 2020-2-21

Reporting Summary

Nature Research wishes to improve the reproducibility of the work that we publish. This form provides structure for consistency and transparency in reporting. For further information on Nature Research policies, see Authors & Referees and the Editorial Policy Checklist.

Statistics

For all statistical analyses, confirm that the following items are present in the figure legend, table legend, main text, or Methods section.

n/a	Confirmed	
☒	☐	The exact sample size (n) for each experimental group/condition, given as a discrete number and unit of measurement
☐	☒	A statement on whether measurements were taken from distinct samples or whether the same sample was measured repeatedly
☒	☐	The statistical test(s) used AND whether they are one- or two-sided<br>*Only common tests should be described solely by name; describe more complex techniques in the Methods section.*
☒	☐	A description of all covariates tested
☒	☐	A description of any assumptions or corrections, such as tests of normality and adjustment for multiple comparisons
☒	☐	A full description of the statistical parameters including central tendency (e.g. means) or other basic estimates (e.g. regression coefficient) AND variation (e.g. standard deviation) or associated estimates of uncertainty (e.g. confidence intervals)
☒	☐	For null hypothesis testing, the test statistic (e.g. F, t, r) with confidence intervals, effect sizes, degrees of freedom and P value noted<br>*Give P values as exact values whenever suitable.*
☒	☐	For Bayesian analysis, information on the choice of priors and Markov chain Monte Carlo settings
☒	☐	For hierarchical and complex designs, identification of the appropriate level for tests and full reporting of outcomes
☒	☐	Estimates of effect sizes (e.g. Cohen's d, Pearson's r), indicating how they were calculated

Our web collection on statistics for biologists contains articles on many of the points above.

Software and code

Policy information about availability of computer code

Data collection	No custom software or computer code was used.
Data analysis	No custom software or computer code was used.

For manuscripts utilizing custom algorithms or software that are central to the research but not yet described in published literature, software must be made available to editors/reviewers. We strongly encourage code deposition in a community repository (e.g. GitHub). See the Nature Research guidelines for submitting code & software for further information.

Data

Policy information about availability of data

All manuscripts must include a data availability statement. This statement should provide the following information, where applicable:

- Accession codes, unique identifiers, or web links for publicly available datasets
- A list of figures that have associated raw data
- A description of any restrictions on data availability

Sequence data that support the findings of this study have been deposited in the DNA Data Bank of Japan (DDBJ) with the accession codes LC524012 to LC524122.

Field-specific reporting

Please select the one below that is the best fit for your research. If you are not sure, read the appropriate sections before making your selection.

☐ Life sciences ☐ Behavioural & social sciences ☒ Ecological, evolutionary & environmental sciences

For a reference copy of the document with all sections, see nature.com/documents/nr-reporting-summary-flat.pdf

Ecological, evolutionary & environmental sciences study design

All studies must disclose on these points even when the disclosure is negative.

Study description	Microbial life in basaltic basement of oceanic crust was investigated according to basalt formation ages (13, 33.5 and 104Ma).
Research sample	We obtained drill core samples during IODP exp. 329 by performing contamination check with fluorescent microspheres. Two depths of 104 Ma site and one depth of 33.5 Ma site were analyzed.These samples were selected based on our previous study (Yamashita et al. 2019). Instead of increasing sample numbers, fractures and veins in the rock samples were analyzed at multiple locations by various analytical techniques.
Sampling strategy	As we targeted microbial life depending on chemical energy from rock-water interactions rather than photosyntheic energy, drilling sites in South Pacific Gyre with minimal primary production at the sea surface was selected.
Data collection	Fluorescence microscopy, µ-Raman Spectroscopy, FT-IR, NanoSIMS, HAADF-STEM, 16S rRNA amplicon analysis.
Timing and spatial scale	October 9 through December 13, 2010. IODP Sites U1365, U1367 and U1368. These sites were chosen based on formation age, thickness of overlying sediments and sea surface primary production.
Data exclusions	Based on Yamashita et al. (2019), samples without smectite clay formation were excluded, because the clay formation indicates ongoing rock-water interactions to support microbial life.
Reproducibility	By multiple analytical techniques were used for the validity of the experimental data.
Randomization	Among shipboard scientists, samples were allocated based on their interest and expertise.
Blinding	Blinding was performed for all analytical techniques without samples.

Did the study involve field work? ☒ Yes ☐ No

Field work, collection and transport

Field conditions	During drilling, sea conditions were good without any rainfall. Temp was around 20 degrees celsius.
Location	U1365: 23.8508°S, 165.6442°W; 5697 m (water depth), U1367: 26.4816°S, 137.9394°W; 4284 m and U1368: 27.9167°S, 123.1609°W; 3738m
Access and import/export	Samples were collected from international water and imported without any quarantein.
Disturbance	Drilling disturbance was not avoided, but the extent of disturbance was evaluated by using microspheres for microbial contamination.

Reporting for specific materials, systems and methods

We require information from authors about some types of materials, experimental systems and methods used in many studies. Here, indicate whether each material, system or method listed is relevant to your study. If you are not sure if a list item applies to your research, read the appropriate section before selecting a response.

Materials & experimental systems

n/a	Involved in the study
☒	☐ Antibodies
☒	☐ Eukaryotic cell lines
☒	☐ Palaeontology
☒	☐ Animals and other organisms
☒	☐ Human research participants
☒	☐ Clinical data

Methods

n/a	Involved in the study
☒	☐ ChIP-seq
☒	☐ Flow cytometry
☒	☐ MRI-based neuroimaging
