# Supplementary material for: Deep microbial proliferation at the basalt interface in 33.5–104 million-year-old oceanic crust
Source: Commun Biol. 2020 Apr 2;3:136. doi: 10.1038/s42003-020-0860-1 (PMC7118141; doi:10.1038/s42003-020-0860-1)
Supplement: Supplementary file 6 — Description of Additional Supplementary Files [file 42003_2020_860_MOESM6_ESM.pdf]

Description of Additional Supplementary Files

File Name: **Supplementary Data 1**

Description: Phylogenetic affiliation and distribution of the highly contaminated sample U1368F-7R3, the drilling-fluid sample from U1365E, and the negative control used for laboratory manipulations based on 16S rRNA gene sequences. The distributions of OTUs detected from the three samples are also shown for the core samples with undetected microspheres.

File Name: **Supplementary Data 2**

Description: Phylogenetic affiliation and distribution of OTUs from the core samples with undetected microspheres based on 16S rRNA gene sequences. Closest cultivated species were retrieved from GenBank through BLASTn searches.
